# Application of enhanced recovery after surgery in total knee arthroplasty in patients with haemophilia A: A pilot study

**DOI:** 10.1002/nop2.605

**Published:** 2020-08-28

**Authors:** Yan Wu, Haipeng Xue, Wenqiang Zhang, Yuhong Wu, Yanwei Yang, Hong Ji

**Affiliations:** ^1^ Bone & Joint Surgery Department Shandong Provincial Qianfoshan Hospital Shandong University Jinan China; ^2^ Department of Orthopedics Affiliated Hospital of Shandong University of Traditional Chinese Medicine Jinan China; ^3^ Oncological Radiotherapy Department Shandong Provincial Qianfoshan Hospital Shandong University Jinan China; ^4^ Quality Control Office The First Affiliated Hospital of Shandong First Medical University Jinan China

**Keywords:** enhanced recovery after surgery, haemophilia A, nursing, rehabilitation, total knee arthroplasty

## Abstract

**Aim:**

To identify the effect of enhanced recovery after surgery (ERAS) and rapid rehabilitation concepts on the outcomes of patients with haemophilia A undergoing total knee arthroplasty.

**Design:**

Randomized controlled trial.

**Methods:**

The primary endpoint was postoperative hospital stay. The secondary endpoints were pain scores, joint function scores, haemoglobin levels at 3 and 7 days after surgery and satisfaction with hospitalization.

**Results:**

Thirty‐two patients were enrolled. Compared with the routine nursing group, the ERAS group showed shorter postoperative hospital stay (14.2 *SD* 0.8 vs. 16.6 ± 1.3 days, *p* < .001), smaller amounts of blood transfusion (924 *SD* 317 vs. 1,263 *SD* 449 ml, *p* = .020) and coagulation factors (37,325 *SD* 5,996 vs. 48,475 *SD* 8,019 U, *p* < .001), lower pain scores at 3 (3.3 *SD* 0.7 vs. 4.3 *SD* 0.7, *p* = .002) and 7 (2.3 SD 0.7 vs. 2.8 ± 0.5, *p* = .015) days, lower hospital for special surgery knee scores at 3 (59.9 *SD* 7.8 vs. 53.6 SD 5.9, *p* = .016) and 7 (77.9 *SD* 6.9 vs. 71.1 ± 7.1, *p* = .009) days and higher satisfaction with hospitalization (94.3 *SD* 1.4 vs. 92.7 *SD* 1.6, *p* = .004).

## 1 INTRODUCTION

Haemophilia is a disease caused by a deficiency in factor VIII (FVIII) or factor IX (FIX) (haemophilia B) (Konkle, Huston, & Nakaya Fletcher, 1993b). The incidence of haemophilia A is 1/5000–1/10000 (Berntorp & Shapiro, 2012; Franchini, Favaloro, & Lippi, 2010; Konkle et al., 1993a, 1993b; Srivastava et al., 2013) and that of haemophilia B is 1/20,000 (Konkle et al., 1993b). Haemophilia A is an X‐linked recessive genetic disease and almost all patients are male (Konkle et al., 1993b; Srivastava et al., 2013).

## 2 BACKGROUND

The knee joint is the most common bleeding site in patients with haemophilia (Konkle et al., 1993b; Srivastava et al., 2013). Intra‐articular bleeding leads to chronic synovitis, articular cartilage damage, peripheral tissue fibrosis, bone destruction, joint pain and joint deformity (Rodriguez & Hoots, 2010; Srivastava et al., 2013). Total knee arthroplasty (TKA) in patients with haemophilia could significantly relieve joint pain, correct joint deformity and improve joint function (Srivastava et al., 2013).

Because of the repeated bleeding in the joints, joint pain, deformity and muscle atrophy are severe in patients with haemophilic knee arthritis; loss of function is also severe; and postoperative rehabilitation is difficult (Lobet et al., 2008; Solimeno & Pasta, 2017; Wiedel, Styabler, Geraghty, & Funk, 2010). Enhanced postoperative recovery (ERAS) is the application of methods that promote postoperative recovery of patients and aims to reduce surgical stress response and complications, improve surgical safety and increase patient satisfaction, thereby accelerating recovery (Chiung‐Jui Su et al., 2015; Rahman & Oussedik, 2015; Van Egmond, Verburg, & Mathijssen, 2015). ERAS is widely used in patients undergoing joint surgery (Frassanito et al., 2019; Soffin & YaDeau, 2016; Vendittoli et al., 2019); however, for patients with haemophilia undergoing TKA, the guidelines or expert consensus on rapid perioperative rehabilitation is lacking and the construction and application of continuing care models are rarely reported.

Thus, this study aimed to apply ERAS and rapid rehabilitation concepts to the care of patients with haemophilia A undergoing TKA to improve the aspects of intraoperative analgesia and haemostasis, dose and duration of perioperative coagulation factor substitutes, selection of postoperative analgesia methods and functional training and recovery timing. This study also aimed to analyse the short‐term effects of this new nursing method.

## 3 METHODS

### 3.1 Study design and patients

This randomized controlled pilot trial was performed at the Department of Joint Surgery, the First Affiliated Hospital of Shandong First Medical University, Jinan, China, which is a large tertiary comprehensive hospital approved by the National Ministry of Public Health with 2,880 open beds). We included all patients with haemophilia A who underwent knee arthroplasty only (including single TKA and double TKA) and met the inclusion criteria for the experimental study. We excluded those who underwent hip arthroplasty. This study was approved by the ethics committee of the First Affiliated Hospital of Shandong First Medical University. All participants signed an informed consent form based on the ethical code of the World Medical Association (Helsinki Declaration); they could withdraw from the study at any time without any penalty. This study was registered on 1 January 2020 (Trial Registration No.: ChiCTR2000029284).

The inclusion criteria were as follows: (a) definitive diagnosis of haemophilia A combined with osteoarthritis stage IV (Luck et al., 2004) requiring TKA; (b) ≥18 years old; (c) preoperative negative coagulation factor inhibitor; (d) clear consciousness, normal communication skills and good understanding; and (e) voluntarily participated in the study and signed the informed consent form. The exclusion criteria included the following: (a) cardiovascular or cerebrovascular diseases; (b) mental disorders; and (c) inability to cooperate.

### 3.2 Grouping

Patients were stratified according to unilateral/bilateral TKA and randomly divided using the random number table prepared by a third‐party biostatistician. All patients underwent surgery under general anaesthesia, which was performed by the same chief physician with 30 years of experience in joint surgery. The corresponding nursing measures were implemented by a team or nurses with 10 years of joint surgery experience.

### 3.3 Routine nursing

Eating and drinking were forbidden starting at 00:00 on the day of the surgery. Drinking could be resumed at 4 hr and eating at 6 hr postoperatively. A tourniquet (20–54–612, VBM Medizintechnik GmbH, Necktar, Germany) was used during TKA. TKA was performed routinely. An automatic analgesia pump was provided. An indwelling drainage tube was placed during the operation. Postoperative analgesia was provided with intravenous infusion of analgesic (0.9% NaCl + flurbiprofen ester 50 mg) (Beijing Tide Pharmaceutical Co., Ltd.) every 12 hr. In patients with a visual analogue scale (VAS) pain score ≥ 6 points, pethidine hydrochloride or morphine was intravenously administered following the physician's advice.

The coagulation factor level was controlled at 100% on the day of surgery and at 40%–60% during the perioperative period. Postoperative activity and functional exercises were performed as follows: the activities were mainly in bed on the day of the surgery, which included isotonic contraction of the quadriceps and hamstring muscles under the protection of a knee brace and on the first day after surgery, when ankle pumping and passive knee pressing were added. On the second day after the surgery, the patients started to get out of bed with assistance. From the second day to the second week after surgery, the patients had to focus on range of motion (ROM) of the knee joint, including active training and auxiliary training with a passive lower limb joint function exerciser (OptiFlex‐X3, DJO, LLC).

### 3.4 ERAS

The patients were encouraged to eat high‐protein foods, such as eggs, milk, soy products, fish and meat. For patients with malnutrition or poor appetite, protein powder and digestive drugs were provided as supplements (Ibrahim, Khan, Nizam, & Haddad, 2013; Moon et al. , 2014; Schwarzkopf, Russell, Shea, & Slover, 2011). At 6 hr before surgery, the patients could eat solid foods, such as eggs and meat. At 4 hr before surgery, the patients could eat chicken, steamed bread and porridge. At 2 hr before surgery, the patients could drink clear liquids containing carbohydrates.

The patients were admitted to the hospital 3–4 days before surgery to learn the postoperative knee activity training and muscle strength training and the use of rehabilitation equipment, such as the OptiFlex‐X3 passive lower limb joint function exerciser, knee extension brace and walking aid. To avoid the risk of infection, bed urination training was performed at least twice a day so that patients could urinate on the bed after surgery without the need for indwelling catheters.

Thirty minutes before surgery, tranexamic acid 1g (Beijing Shuanghe Pharmaceutical Co., Ltd., National Medicine Zhunzi H11020633) in 100 ml of 0.9% NaCl was infused intravenously. After joint capsule closure, tranexamic acid 1 g in 60 ml of 0.9% NaCl was infused in the articular cavity to reduce bleeding.

A tourniquet was not used during the surgery to prevent postoperative limb swelling and pain. Multipoint administration of a “cocktail” in the subcutaneous, synovial and periosteal soft tissues was performed in lieu of patient‐controlled analgesia pump to reduce adverse reactions, such as gastrointestinal discomfort. Specifically, the cocktail contained the following (Kong et al., 2016): ropivacaine 75 mg (H20100105, AstraZeneca), compound betamethasone 7 mg (H20130188, Schering‐Plough Corporate) and adrenaline hydrochloride 0.1 ml (all diluted in 40 ml of 0.9% NaCl) (H23023237, Harbin Pharmaceutical Group Co., Ltd.).

Pressure dressing and compression on the incision were applied to stop bleeding, and a drainage tube was not indwelled. Intermittent cold compresses were placed on the incision to reduce joint bleeding. Elastic bandages were applied to promote venous return. The patients were enjoined to lift both lower limbs to reduce the swelling of the affected limb.

On the day of the surgery, liquid or semi‐liquid diet was provided. Postoperatively, when the patient was awake and the swallowing function was evaluated to be good, water and food consumption was gradually allowed. Subsequently, an ordinary diet, primarily bland food, was started. The patient was encouraged to take high‐quality proteins (Ibrahim et al., 2013; Moon et al., 2014; Schwarzkopf et al., 2011; Zhou et al., 2016).

Advanced analgesia and multimodal combined analgesia were used instead of an automatically controlled analgesia pump. Flurbiprofen ester 50 mg was intravenously infused every 12 hr, but for no more than 10 days to avoid gastrointestinal bleeding. Buprenorphine transdermal patches (LTS Lohmann Therapie‐Systeme AG andernach) were used to assist analgesia on the day of the surgery. Intermittent cold compresses were placed for 20–30 min according to the patient's tolerance. The nurses paid attention to the patient's nighttime pain; sedative‐hypnotics combined with weak opioid analgesics was administered before bedtime, if necessary, to ensure sleep and reduce nighttime pain. Patients with a VAS pain score ≥ 6 points received pethidine hydrochloride or morphine, which was administered intravenously, following the physician's advice.

Based on the consensus of specialists in China, for the perioperative management of haemophilia in patients who underwent orthopaedic surgery of haemophilia, the FVIII levels should be maintained at 80%–100% on the day of surgery, 80% at 1–3 days after the surgery, 60% at 4–7 days after surgery, 40% at 8–14 days after surgery and 20% at 15–30 days after surgery (Wang et al., 2016). It was calculated by infusing the FVIII preparation (Shondong Taibang Biological Products Col., Ltd.) 1 U per kilogram of body weight to increase the FVIII:C level by about 2%. The required dose (U) was calculated as follows: body weight (kg) × (required FVIII level—actual measured FVIII level)/2. The half‐life of FVIII is 8–12 hr; thus, FVIII was administered every 12 hr. Orthopaedic surgery requires intensive factor replacement therapy to prevent bleeding and tissue trauma to stimulate the immune response, which induces the generation of inhibitors (Shan, Shan, Suzuki, Nouh, & Saxena, 2015). On day 7 postoperatively or when haemostasis was not good, coagulation factor inhibitors were examined by the Bethesda method (Torita et al., 2011).

Postoperative activities had to be initiated as soon as possible. After surgery, the muscle strength of the patients was evaluated. When the operated limb muscle strength was below grade III, the patients were encouraged to move in bed. When the operated limb muscle strength reached grade ≥ III and/or the healthy limb muscle strength reached grade ≥ IV, the patient started to get out of the bed with assistance. Early walking and gait training could significantly reduce the abnormal force on the prosthesis. Functional exercise after TKA focused on muscle strengthening and joint mobility improvement. All the training was conducted 0.5–4 hr after the infusion of FVIII preparation. From the day of the surgery to 1 day postoperatively, the exercise was primarily isometric exercise of the quadriceps and hamstring muscles, which was performed on the bed mainly by knee extension training. At 1–4 days after surgery, the exercise focused on isometric and isotonic exercises of quadriceps and hamstring muscles and ROM training of the knee joint. Thus, the main training methods included knee extension training, straight leg raising training and knee‐bending training on the bed, bedside and benches. For patients with severe deformity, wax therapy was added on the fifth day after surgery and the OptiFlex‐X3 passive lower limb joint function exerciser was used after the wax therapy. The initial degree referred to the actual joint activity of the patient. The angle was then increased by 10° increments depending on patients' tolerance. The exerciser was used twice a day (30 min each session).

Patients who met the following criteria were discharged: (a) no active bleeding in the joint cavity and no haematoma formation around the knee joint; (b) knee joint ROM of 0–90°; (c) healed incision without exudation or infection; and (d) postactivity VAS pain score ≤ 4 (oral analgesics could alleviate the pain).

### 3.5 Evaluation of outcomes

In this study, we assessed the following outcomes: perioperative hospital stay, usage of coagulation factors, pain scores, joint function scores, haemoglobin levels at 3 and 7 days after surgery and satisfaction with hospitalization (evaluated by the patient and his/her family using a 100‐point scoring system). VAS pain scores (range 0–10 points; the higher the score, the more severe the pain) were evaluated after each activity or functional exercise. Joint function was evaluated using the hospital for special surgery (HSS) knee scoring system (Bach et al., 2002), which includes the following aspects: pain, function, joint mobility, muscle strength, knee flexion deformity, knee instability and deduction project. The total score is 100 points (30 points for pain, 22 points for function, 18 points for activity range, 10 points for muscle strength, 10 points for flexion deformity and 10 points for joint stability). An HSS score ≥ 85 is excellent, 70–84 is good, 60–69 is moderate and ≤ 59 is poor. The HSS score was assessed by an associate chief physician who had 15 years of experience in joint surgery.

All data were obtained by two physicians. Different opinions were resolved by senior doctors. The data were entered by two personnel of the research group. For the experiment, laboratory testing and data collection and analysis were performed in a blinded manner.

### 3.6 Statistical analysis

Data were analysed using SPSS 21.0 (IBM). Normally distributed continuous variables were presented as means ± *SD* and analysed using the independent sample *t* test. Continuous variables that were not normally distributed were presented as medians (interquartile ranges) and analysed using non‐parametric tests. Categorical variables were presented as *n* (%) and analysed using chi‐square test or Fisher's exact test, as appropriate. Two‐sided *p* values < .05 were considered statistically significant.

Because of the small sample size and to ensure the accuracy of the results, we included all patients with haemophilia A who underwent knee arthroplasty only (including single TKA and double TKA), thus, a sample size calculation was not performed.

## 4 RESULTS

### 4.1 Characteristics of the patients

Thirty‐two patients were enrolled according to the inclusion criteria (16 patients per group). Twenty underwent bilateral surgery and were randomly assigned to two groups (10 in each group): ERAS and routine nursing groups. Twelve underwent unilateral surgery and were randomly assigned to the two groups (six in each group). None of the 32 patients withdrew or were lost to follow‐up. No differences in the baseline characteristics between the two groups were found (Table 1).

**TABLE 1:** Baseline characteristics of the patients

	ERAS (*n* = 16)	Routine (*n* = 16)	*p*
Age	35.6 ± 6.7	31.7 ± 9.2	.189
Bilateral surgery	6/16 (37.5%)	6/16 (37.5%)	1.000
Weight (kg)	68 ± 13.0	67 ± 11.7	.821
Preoperative FVIII levels (%)	2.2 ± 0.9	2.6 ± 0.9	.220
Preoperative VAS	3.4 ± 0.7	3.4 ± 0.7	1.000
Preoperative HSS	52.0 ± 7.6	51.6 ± 9.3	.885
Preoperative Hb (g/L)	144.3 ± 17.3	149.6 ± 12.6	.329

Abbreviations: ERAS, enhanced recovery after surgery; FVIII, factor VIII; HSS, Hospital for Special Surgery knee score; Hb, haemoglobin; VAS, visual analog scale.

### 4.2 Intraoperative and postoperative indicators

The endpoints are presented in Table 2. The postoperative hospital stay was shorter in the ERAS group than in the routine nursing group (14.2 *SD* 0.8 vs. 16.6 Sd 1.3 days, *p* < .001). Compared with the routine nursing group, the ERAS group had smaller amounts of blood transfusion (924 *SD* 317 vs. 1,263 *SD* 449 ml, *p* = .020) and FVIII preparation (37,325 *SD* 5,996 vs. 48,475 *SD* 8,019 U, *p* < .001). Pain scores at 3 (3.3 *SD* 0.7 vs. 4.3 *SD* 0.7, *p* = .002) and 7 (2.3 *SD* 0.7 vs. 2.8 *SD* 0.5, *p* = .015) days after surgery were lower in the ERAS group than in the routine nursing group. The patients in the ERAS group received analgesics for a total of eight times and those in the routine nursing group used analgesics for a total of 19 times. The HSS scores at 3 (59.9 *SD* 7.8 vs. 53.6 *SD* 5.9, *p* = .016) and 7 (77.9 *SD* 6.9 vs. 71.1 *SD* 7.1, *p* = .009) days after surgery were lower in the ERAS group than in the routine nursing group. Satisfaction with hospitalization was higher in the ERAS group (94.3 *SD* 1.4 vs. 92.7 *SD* 1.6, *p* = .004). No significant differences in haemoglobin levels between the two groups at 3 (*p* = .291) and 7 (*p* = .296) days after surgery were found.

**TABLE 2:** Intraoperative and postoperative indexes

	ERAS (*n* = 16)	Routine (*n* = 16)	*p* value
Postoperative hospital stays (days)	14.2 ± 0.8	16.6 ± 1.3	<.001
Blood transfusion volume (ml)	924.0 ± 317.3	1,262.5 ± 448.5	.020
Amount of FVIII usage (U)	37,325 ± 5,996	48,475 ± 8,019	<.001
VAS at day 3	3.3 ± 0.7	4.3 ± 0.7	.002
VAS at day 7	2.3 ± 0.7	2.8 ± 0.5	.015
Use of analgesic (total numbers of times in all patients)	8	19	—
Hb at day 3 (g/L)	93.0 ± 22.1	85.9 ± 14.7	.291
Hb at day 7 (g/L)	102.8 ± 18.8	96.3 ± 15.3	.296
HSS score at day 3	59.9 ± 7.8	53.6 ± 5.9	.016
HSS score at day 7	77.9 ± 6.9	71.1 ± 7.1	.009
Hospital satisfaction (/100)	94.3 ± 1.4	92.7 ± 1.6	.004

Abbreviations: ERAS, enhanced recovery after surgery; FVIII, factor VIII; Hb, haemoglobin; HSS, Hospital for Special Surgery knee score; VAS, visual analog scale.

### 4.3 Complications

Table 3 presents the complications. Joint haematoma occurred in one and three patients in the ERAS and routine nursing groups, respectively. A bleeding event occurred in one patient in the routine nursing group. Unhealed incision was noted in two and six patients in the ERAS and routine nursing groups, respectively. No cases of ligament injury, periprosthetic joint infection, nerve injury, coagulation factor inhibition and deep venous thrombosis were observed in both groups.

**TABLE 3:** Complications

	ERAS (*n* = 16)	Routine (*n* = 16)	*p*
Joint haematoma	1 (6.3%)	3 (18.8%)	.600
Ligament injury	0	0	—
Periprosthetic joint infection	0	0	—
Nerve injury	0	0	—
Coagulation factor inhibition	0	0	—
Bleeding events	0	1 (6.3%)	1.000
Unhealed incision (tension blister around the incision or exudation)	2 (12.5%)	6 (37.5%)	.220
Deep venous thrombosis of lower extremities	0	0	—

## 5 DISCUSSION

Patients with haemophilia develop severe joint conditions that often require joint replacement. ERAS aims at accelerating recovery after surgery. The objective of our study was to apply the ERAS and rapid rehabilitation concepts to the care of patients with haemophilia A undergoing TKA and to analyse the short‐term effects of this new nursing method. The results of this pilot study suggest that ERAS in patients with haemophilia A undergoing knee joint replacement may shorten hospital stay, promote joint function recovery and improve patient satisfaction.

The concept of ERAS and rapid rehabilitation is widely used in patients undergoing TKA for osteoarthritis (Frassanito et al., 2019; Soffin & YaDeau, 2016; Vendittoli et al., 2019). However, no data on the application of ERAS for TKA in patients with haemophilia A exist. Patients with haemophilic knee osteoarthritis have poor preoperative joint function; thus, surgical procedures are complicated and postoperative rehabilitation is difficult (Lobet et al., 2008; Solimeno & Pasta, 2017; Wiedel et al., 2010). In January 2017, our department designed an ERAS protocol for the rapid rehabilitation of patients with haemophilia A undergoing TKA. This pilot study included an intervention tailored to the specific characteristics of haemophilia A patients and achieved good results.

A good nutritional status could increase a patient's tolerance to surgery, whereas perioperative malnutrition could lead to delayed wound healing, increase the risk of incision infection and prolong hospital stay (Ibrahim et al., 2013; Inacio et al., 2014). Moreover, high‐protein intake before and after surgery could enhance the patient's tolerance to not only surgery but also postoperative functional exercise, shorten hospitalization and improve satisfaction with hospitalization (Ibrahim et al., 2013; Inacio et al., 2014). Previous studies also showed that shortening the preoperative fasting time is beneficial in reducing hunger, thirst, irritability, nervousness and other adverse reactions of the patients before surgery and even in shortening hospitalization (Nygren, Thorell, & Ljungqvist, 2015). In this study, the patients in the ERAS group fasted for 4 hr and were forbidden to drink 2 hr before the surgery. Compared with the routine nursing group, the ERAS group had a significantly reduced incidence of hunger, thirst, irritability and nervousness and their satisfaction with hospitalization was significantly improved.

According to the Chinese expert consensus on enhanced recovery after hip and knee joint replacement surgery—perioperative pain and sleep management (Shen et al., 2016), the use of multimodal combined analgesia and good sleep could significantly relieve nighttime pain, facilitate physical recovery and promote early activities and functional exercise, thereby improving patients’ comfort and satisfaction and accelerating full recovery. In the ERAS group, a tourniquet was not employed and a cocktail injection, including local anaesthetic, anti‐inflammatory and vasoconstrictor agents, was used during surgery. After surgery, multimodal analgesia was induced. Compared with the routine nursing group, the ERAS group had a significantly reduced pain score.

If exercises could not be performed early after TKA, a large amount of collagen tissues will quickly deposit around the joints, which in turn causes joint adhesion and limits joint ROM. A previous study showed that new collagen tissues appear on the second day after surgery and reach a peak after 5–7 days (Cheng, Li, & Yu, 2006). Thus, early functional exercise, that is, immediately after the patient awakes from anaesthesia, is recommended. Moreover, active functional exercise is beneficial for the early recovery of joint function and ROM and could reduce related complications (Shan et al., 2015). Compared with the routine nursing group, the ERAS group showed no statistically significant change in haemoglobin levels at 3 and 7 days postoperatively. In addition, joint function at 3 and 7 days after surgery was significantly better in the ERAS group than in the routine nursing group. Such good outcomes, which are associated with early exercise, were observed in patients with osteoarthritis in previous studies (Frassanito et al., 2019; Soffin & YaDeau, 2016; Vendittoli et al., 2019). In our study, patients with haemophilia A require a specific coagulation factor management in order not to aggravate bleeding. Nevertheless, as our study is a pilot proof‐of‐concept study, confirmation by multicentre trials is warranted.

This study has some limitations. Firstly, as this was a single‐centre study, the sample size is small and thus the prevalence of haemophilia A in the population is low. In addition, the study only assessed hospitalization indicators. Secondly, the operation and nursing measures in the two groups were performed by the same team. The surgeons and the nurses who implemented the nursing interventions were not blinded to the study's objective, which possibly affected the results. Thirdly, haemophilia A arthritis usually involves multiple joints and some patients received TKA and total hip arthroplasty (THA; artificial hip replacement) simultaneously. However, we selected patients who had TKA only in this study. We could not verify the effect of ERAS on patients with haemophilia A who underwent both TKA and THA; thus, information on the application of ERAS on such patients is limited. Hence, further large‐scale, multicentre, long‐term, randomized controlled clinical trials to verify the effect of ERAS on patients with haemophilia who underwent TKA are needed. Moreover, future research to investigate the effect of ERAS on patients with haemophilia A who underwent TKA and THA simultaneously and on patients who received THA alone is warranted.

## 6 CONCLUSIONS

Compared with routine nursing interventions, the implementation of ERAS may promote recovery of joint function and shorten the length of hospital stays among patients with haemophilia A undergoing TKA. Patient satisfaction was higher in the ERAS group, and no complications were noted.

## RELEVANCE TO CLINICAL PRACTICE

Compared with routine nursing, the implementation of ERAS in patients with haemophilia A undergoing TKA could promote joint function recovery and shorten the length of hospital stays.

## CONFLICT OF INTEREST

The authors declare that they have no competing interests.

## AUTHOR CONTRIBUTIONS

HJ: Carry out the studies, participation of collecting data and manuscript draft. YW and WQZ: Performance of patient assessment and data collection. HPX and YHW: gave the professional technical guidance during the implementation of the project.WYY: Performance of statistical analysis and entry. All authors read and approved the final manuscript.

## Data Availability

Research data used to support the findings of this study are available from the corresponding author upon request. Or research data can be found online at: http://www.medresman.org.cn/pub/en/proj/search.aspx
